# Development of a Sustainable Simulator and Simulation Program for Laparoscopic Skills Training in Haiti

**DOI:** 10.7759/cureus.632

**Published:** 2016-06-05

**Authors:** Emile Damas, Chesnel Norcéide, Yvel Zephyr, Kerry-Lynn Williams, Tia Renouf, Adam Dubrowski

**Affiliations:** 1 Surgery Department, Justinien University Hospital; 2 Paediatrics, Justinian University Hospital; 3 Department of OBGYN, Director of Training and Research, Justinien University Hospital; 4 Faculty of Medicine, Memorial University of Newfoundland; 5 Emergency Medicine, Memorial University of Newfoundland; 6 Emergency Medicine, Pediatrics, Memorial University of Newfoundland; 7 Marine Institute, Memorial University of Newfoundland

**Keywords:** laparoscopic, haiti

## Abstract

Laparoscopic surgery has been shown to have many favorable effects on surgical outcomes and postoperative recovery times. However, the cost of currently available training programs, such as the Fundamentals of Laparoscopic Surgery (FLS), limits their adoption in developing countries. To address this cost constraint, educators at the Justinian University Hospital (JUH) in Northern Haiti used local materials to build their own laparoscopic skills box trainer. This trainer is used to teach all surgical and OB/GYN residents in their laparoscopic skills program. The progressive curriculum consists of seven modules, three of which are for all trainees and four of which are specifically for surgery and OB/GYN (2). The seven modules are arranged in the order of difficulty; they start with basic maneuvers and progress to complex skills. This report describes both the preparation of the seven models and evaluation of the skills that are learned. This approach may facilitate global access to feasible, progressive, and sustainable laparoscopic training.

## Introduction

Laparoscopic surgery has many advantages for patient care, chiefly, minimal trauma to tissues and faster postoperative recovery times. It is a common technique in developed countries. However, it been only minimally adopted in developing countries, like Haiti, despite the availability of telesimulation for training laparoscopic surgery teachers [1-8]. Fundamentals of Laparoscopic Surgery (FLS) is, for example, one well-established and validated example of telesimulation that was successfully implemented in Botswana. Using SKYPE, an FLS-certified trainer in Canada was able to supervise FLS training sessions in that country. Though in many ways a feasible solution, access to equipment was problematic. That is, the trainees in Botswana were required to use FLS-certified trainers and exercises; these are prohibitively expensive in some contexts.

The Justinian University Hospital (JUH) is Northern Haiti’s primary teaching institution. It trains its residents in laparoscopic surgery at Milot and Pignon, two nearby hospitals that perform the technique. It was determined that simulation-based education at the JUH would help to prepare residents to further this laparoscopic work in Milot and Pignon.

This report describes a feasible approach to laparoscopic skills training, developed at JUH for all their surgical and OB/GYN residents. Educators there used local materials to build a box trainer, like the FLS trainer, in order to provide accessible education for trainers and students working in that resource-poor region. The program uses the progressive simulation training approach described by Cristancho, et al. [9-10], along with validated assessment methods. With progressive simulation, a given complex procedure is deconstructed into its subcomponents (skills), which are then further deconstructed to *their* subcomponents (tasks). This is a hierarchical training approach; only after the learner becomes proficient at skills can he or she begin to practice tasks. They can practice the entire procedure once they have developed task proficiency. Learning objectives are written for these skills, tasks, and procedures; they consequently inform the types of simulation used both for practice and assessment.

## Technical report

### Box trainer

**Figure 1:**
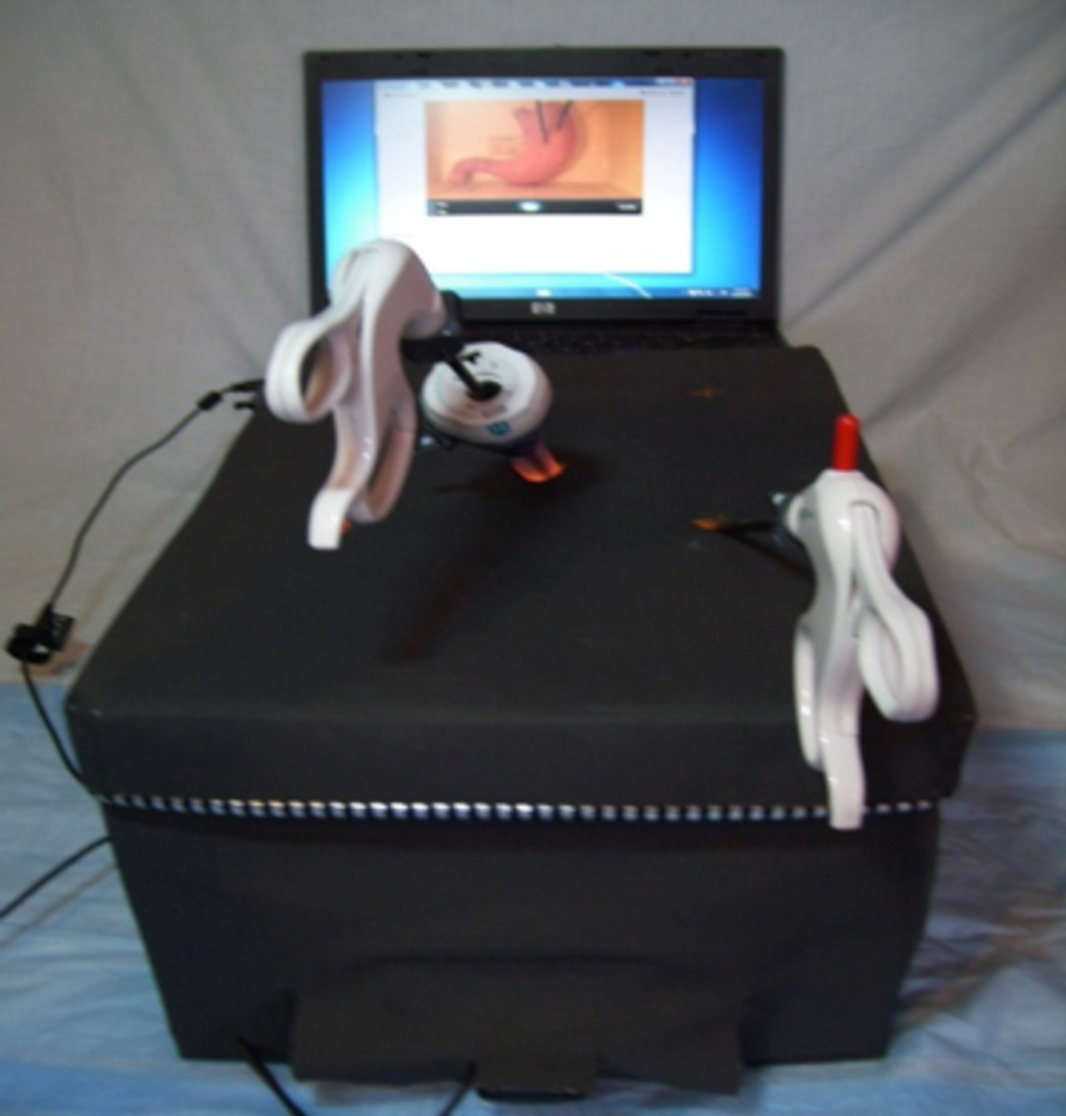
Laparoscopic box trainer.

Dr. Damas Emile from the JUH developed the laparoscopic box trainer (Figure 1). The outer shell is constructed from cardboard and is covered with plastic. There are five ports on the top surface and two laterally. The interior of the box trainer is illuminated with a standard 15 W lamp. A Logitech HD Webcam C270 web camera (Logitech, Newark, CA), placed at the back of the trainer and connected to a laptop computer, serves as a display. Alternatively, for classroom-type viewing, the computer can be connected to a different display device, such as an LCD projector or screen.

### Curriculum

The simulation-based laparoscopic skills curriculum for senior surgical and OB/GYN residents is based on progressive training. It consists of seven modules, three of which are shared amongst all trainees; the other four are specifically for surgery (two) and OB/GYN (two). The seven modules are arranged in order of difficulty, starting with basic maneuvers and progressing to more specific, complex skills (Table 1).

**Table 1:** Summarized Proposed Progressive Laparoscopic Skills Curriculum

Training Level	Training Module	Objectives	Assessment
Basic skills	Peg transfer Cutting Suturing and foam tying on a foam pad	Learn basic maneuvers Hand-eye coordination Working in 3D space using 2D displays	Time Precision Global Rating Scales
Complex skills	Cholecystectomy Pyloroplasty Ectopic pregnancy Ovarian cystectomy	For each of the skills, the objective is to learn all necessary skills and optimize the flow of performance (become proficient).	Global Rating Scales Appropriate task-specific checklists

Basic skills, like hand-eye coordination and manipulating three-dimensional (3D) space using two-dimensional (2D) displays, are taught in Modules 1–3. This is accomplished by practicing general manual skills, such as peg transfer, cutting, suturing, and knot tying. Modules 4–7 progress to more complex procedures. Surgical learners move to a cholecystectomy module (Module 4), and pyloroplasty (Module 5), whereas OB/GYN learners progress to a simulated ectopic pregnancy module (Module 6) and a simulated ovarian cystectomy (Module 7). 

Learning objectives and metrics are provided for each simulation module. For the basic skills (Modules 1-3), the metrics are either time or precision; for the complex procedures (Modules 4-7), a Global Rating Scale (GRS) (Table 2: modified from Reznick, et al.) [11-13] and task-specific checklists are used (refer to sections below for sample of checklists for each module).

**Table 2:** Objective Structured Assessment of Technical Skills “1” indicates worst possible score and “5” indicates best possible score

Performance Characteristic	Rating				
Depth perception	1	2	3	4	5
	Constantly overshoots target, wide swings, slow to correct		Some overshooting or missing of target, but quick to correct		Accurately directs instruments in the correct plane to target
Bimanual dexterity	1	2	3	4	5
	Uses only one hand, ignores nondominant hand, poor coordination between hands		Uses both hands but does not optimize interaction between hands		Expertly uses both hands in a complementary manner to provide optimal exposure
Efficiency	1	2	3	4	5
	Uncertain, inefficient efforts, many tentative movements, constantly changing focus or persisting without progress		Efficient progress but some unnecessary movements		Confident, efficient, and safe conduct, maintains focus on task until it is better performed via an alternate approach
Tissue handling	1	2	3	4	5
	Frequent use of unnecessary force, tears tissues, injures adjacent structures, poor grasper control, grasper frequently slips		Handles tissue reasonably well, minor trauma to adjacent tissue (i.e. occasional unnecessary bleeding or slipping of the grasper)		Handles tissue well, applies appropriate traction, negligible injury to adjacent structures
Autonomy	1	2	3	4	5
	Unable to complete entire task, even with verbal guidance		Able to complete task safely with moderate guidance		Able to complete task independently without prompting
Instrument handling	1	2	3	4	5
	Repeatedly makes tentative or awkward/jerky movements		Competent use of instruments but occasionally appeared stiff or awkward		Fluid moves with instruments and no awkwardness

### Module 1: Peg transfer

**Figure 2:**
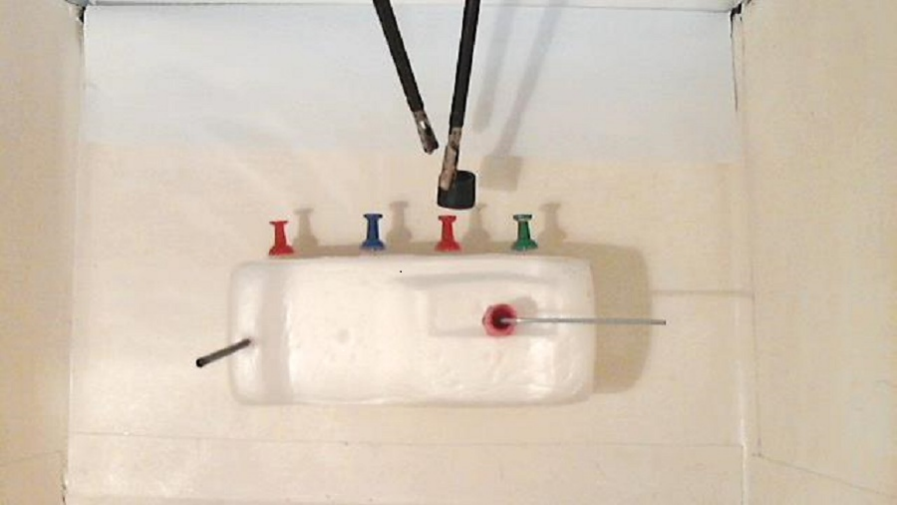
Laparoscopic peg transfer.

The residents’ learning objective here is to develop laparoscopic dexterity. They will also learn how to work within 3D space using 2D displays by manipulating the laparoscopic graspers, and by practicing independently until they reach a predefined level of proficiency [14-15]. Performance, as it relates to proficiency, is measured by timing (time spent) and precision (number of pegs dropped) (Figure 2).

### Module 2: Cutting

**Figure 3:**
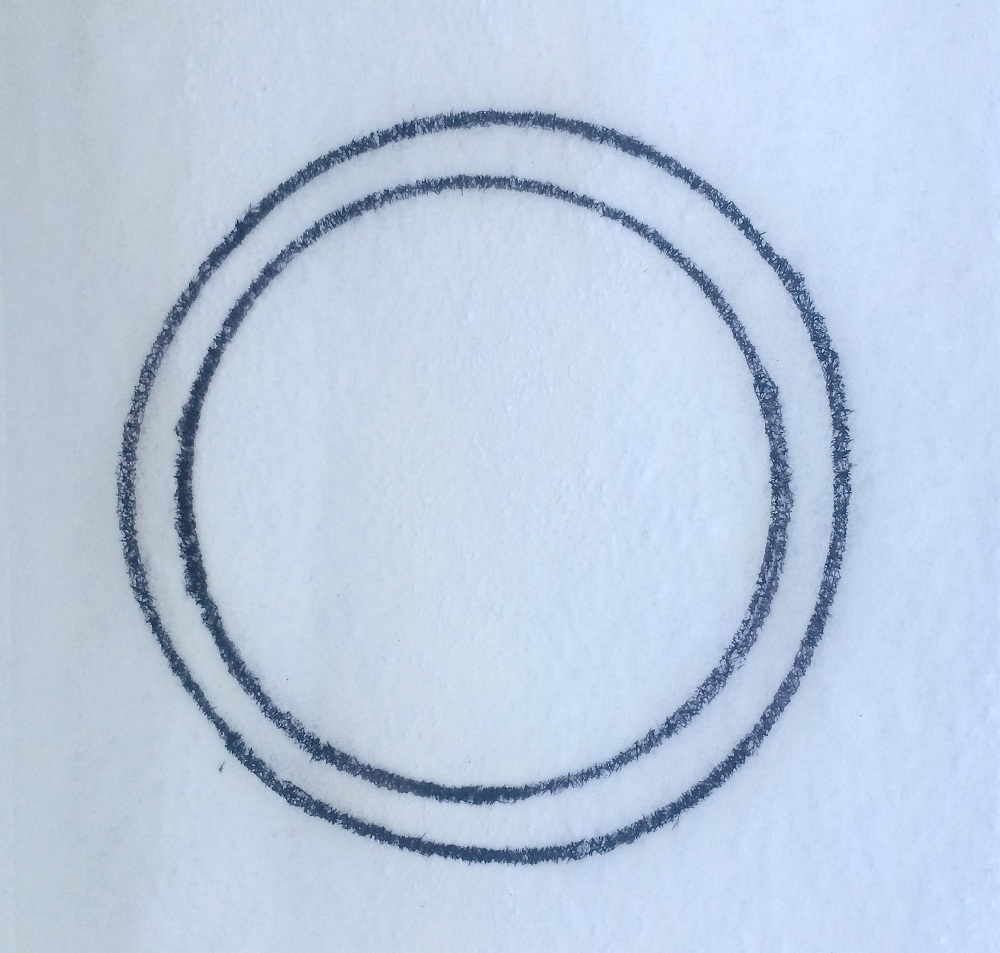
Laparoscopic cutting.

A circular pattern is cut along a 1 mm-wide line (Figure 3). The residents’ learning objectives here are again to develop laparoscopic dexterity and to work within a 3D space using 2D displays while using laparoscopic scissors. Residents practice independently until they reach the predefined proficiency as in Module 1.

### Module 3: Suturing and knot tying on a foam pad

**Figure 4:**
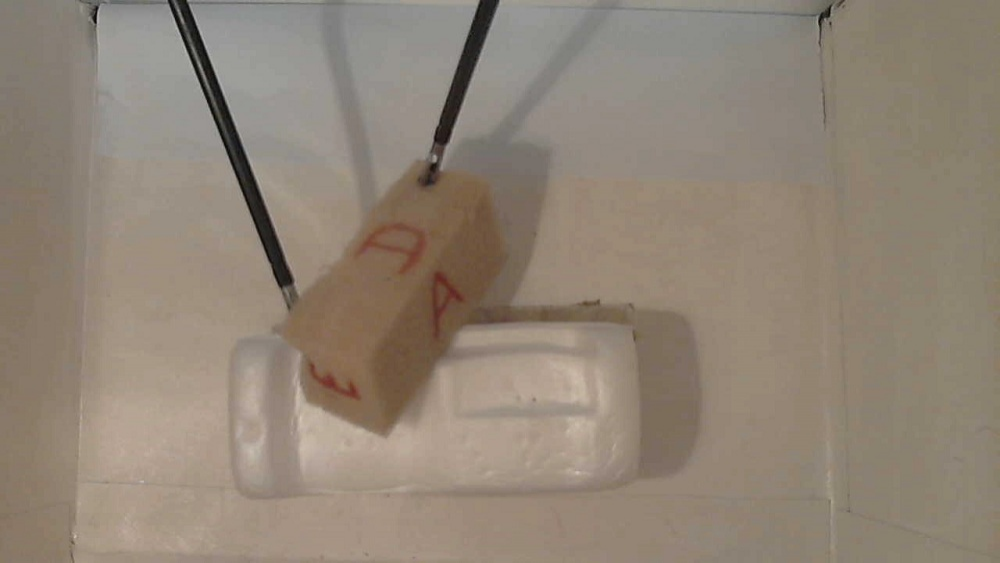
Suturing and knot tying on a foam pad.

Learning objectives for this module are the same as above (to develop laparoscopic dexterity and to work within 3D space using 2D displays) as well as to acquire basic laparoscopic suturing and knot tying skills. This is accomplished by placing a strip of foam (20 cm × 5 cm) in the trainer (Figure 4). The goal for residents is to make a tube out of the rectangular foam by suturing the two edges of the foam together.

An experienced trainer supervises the practice sessions during this module. The trainer can provide both formative and summative feedback based on the GRS (Table 2) and skill-specific checklist (Table 3) adapted from Reznick, et al. [11, 16].

**Table 3:** Task-Specific Checklist Adapted from Reznick, et al.

Item		Not Done or Incorrect	Done Correctly
1	Selects appropriate needle driver and sutures	0	1
2	Needle loaded from 1/2 to 2/3 from tip	0	1
3	Uses laparoscopic needle holder and forceps to handle needle	0	1
4	Needle enters tissues at right angles (80% of bites)	0	1
5	Single attempt at needle passage through tissues (90% of bites)	0	1
6	Follow through on curve of needle on entrance (80% of bites)	0	1
7	Follow through on curve of needle on exit (80% of bites)	0	1
8	Minimal damage with forceps	0	1
9	Equal suture spacing	0	1
10	Equal bites on each side (80% of bites)	0	1
11	Square knots (minimum three throws on knots)	0	1
12	Suture cut to appropriate length (does not interfere with next stitch)	0	1
13	Apposition of tissues without excessive tension on suture	0	1
14	Appropriate alignment of tissues (no torsion)	0	1
Maximum total score, Total score			(14)

### Module 4: Cholecystectomy

**Figure 5:**
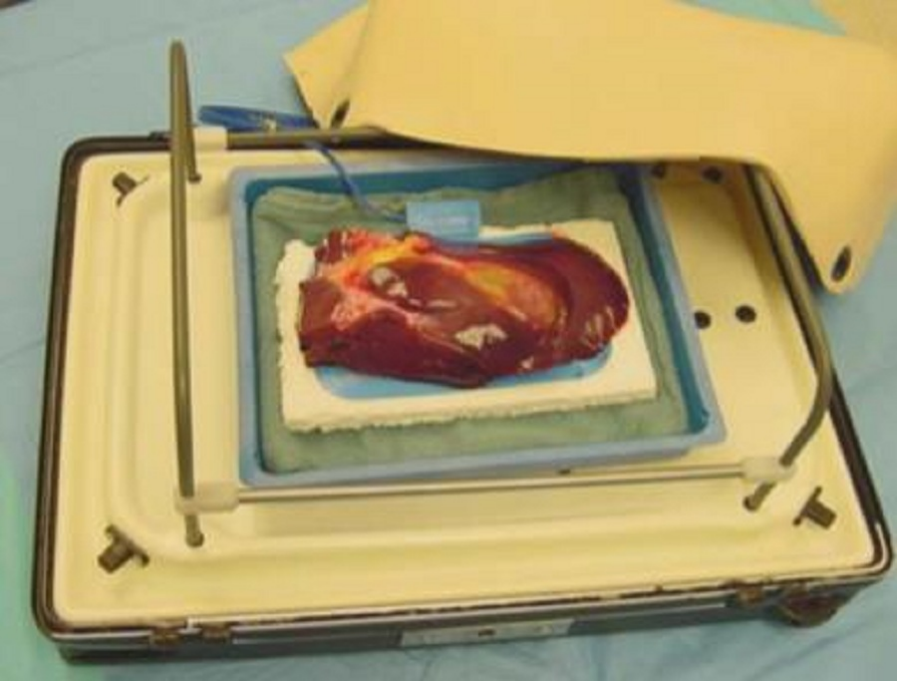
Cholecystectomy lap trainer.

The residents’ learning objectives here are to develop specific skills for performing a cholecystectomy on an animal model. This is accomplished by placing a porcine liver and a gall bladder (obtained from an abattoir) inside the box trainer (Figure 5). While positioned upside down, the extra liver is removed to ensure that the gall bladder, bile duct, and vessels are intact (this should be approximately 15–20 cm × 25–30 cm). The specimen is fixed to a cautery pad and positioned on a piece of Styrofoam™ (Dow Chemical Co., Midland, MI, USA) while the margins of the liver are stapled to the Styrofoam™ in order to prevent it from being lifted up during instrumental manipulation. Finally, the secured specimen is placed on a shallow tray and the tray is positioned within the box trainer.

An experienced trainer supervises these sessions and can provide feedback based on the GRS (Table 2) and skill-specific checklist (Table 4) adapted from Vassiliou, et al. [17]. 

**Table 4:** Task-Specific Checklist: Dissection of the Gallbladder from the Liver Bed Adapted from Vassiliou, et al.

Item		Not Done or Incorrect	Done Correctly
1	Selects appropriate needle driver and sutures	0	1
2	Needle loaded from 1/2 to 2/3 from tip	0	1
3	Uses cautery only when all conducting areas are in field of view	0	1
4	Has good control of the instrument, minimizes recoil	0	1
5	Grasps gallbladder near clips to begin dissection	0	1
6	Readjusts tension on gallbladder to optimize exposure	0	1
7	Avoids dissecting into liver, causing undue bleeding	0	1
8	Avoids perforation of the gallbladder	0	1
9	Avoids spillage of gallstones	0	1
10	Maximizes useful dissection in one area before changing approach	0	1
11	Performs dissection in the appropriate plane the majority of the time	0	1
12	Obviates the need for surgeon takeover	0	1
Maximum total score, Total score			(12)

### Module 5: Pyloroplasty

**Figure 6:**
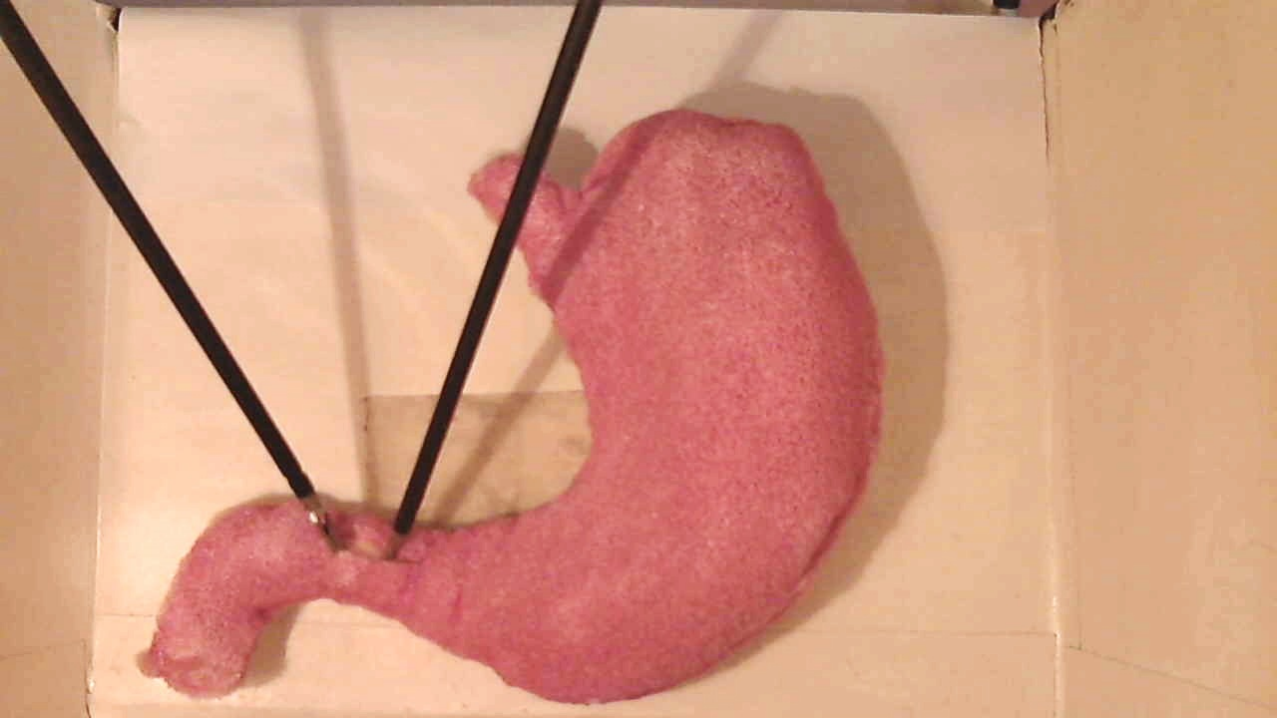
Pyloroplasty lap trainer.

The residents' learning objectives here are to develop the necessary specific skills to perform a pyloroplasty on an inanimate model. A stomach is made with sponge and colored appropriately. An inflated condom is introduced into the stomach to increase its volume. The stomach is then placed in the box trainer (Figure 6). 

An experienced trainer supervises the practice sessions at this module and can provide feedback based on the GRS (Table 2) and skill-specific checklist (Table 5). 

**Table 5:** Task-Specific Checklist: Pyloroplasty

Item		Not Done or Incorrect	Done Correctly
1	Palpates extent of pylorus	0	1
2	Perpendicular (non-scythed) entry into stomach	0	1
3	Atraumatic entry to stomach	0	1
4	Adequate length to encompass pylorus (minimum 3 cm)	0	1
5	Stay sutures held with snaps	0	1
6	Selects appropriate needle driver and suture	0	1
7	Needle loaded 1/2 to 2/3 from tip	0	1
8	Index used to stabilize needle driver	0	1
9	Needle enters bowel at right angles (80% of bites)	0	1
10	Single attempt at needle passage through bowel (90% of bites)	0	1
11	Follow through on curve of needle on entrance (80% of bites)	0	1
12	Follow through on curve of needle on exit (80% of bites)	0	1
13	Forceps used on seromuscular layer of bowel only majority of time	0	1
14	Minimal damage with forceps	0	1
15	Uses forceps to handle needle	0	1
16	Suture spacing 3 to 5 mm	0	1
17	Equal bites on each side on 80% of bites	0	1
18	Square knots	0	1
19	Minimum three throws on knots	0	1
20	Suture cut to appropriate length (does not interfere with next stitch)	0	1
21	No mucosal pouting	0	1
22	Apposition of bowel without excessive tension on sutures	0	1
23	Closure accomplished evenly	0	1
Maximum total score, Total score			(23)

### Module 6: Ectopic pregnancy

**Figure 7:**
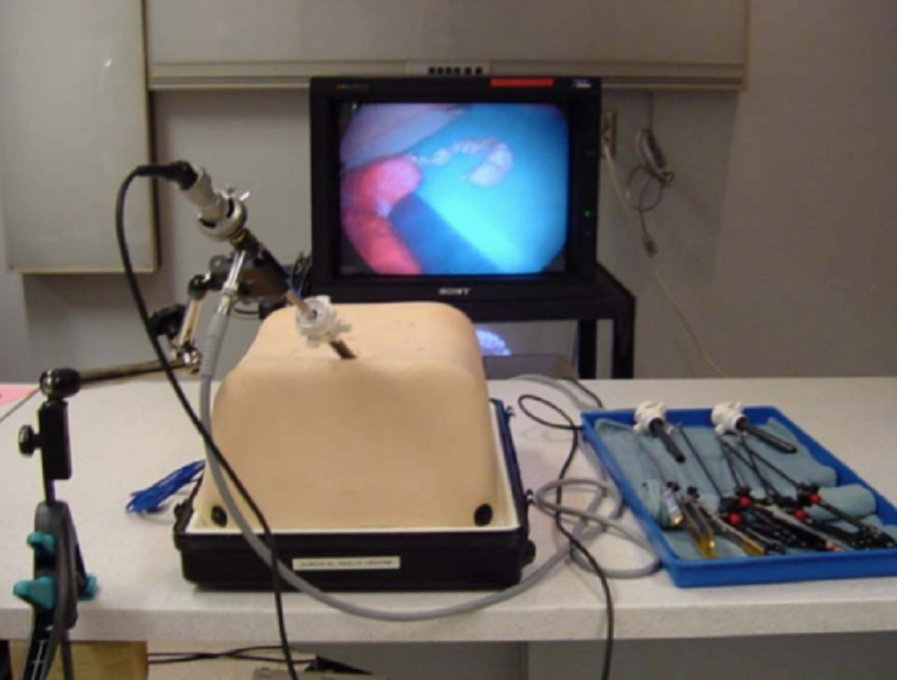
Ectopic pregnancy lap trainer.

The residents’ learning objectives here are to develop specific skills related to ectopic pregnancy. Modeling clay, such as Play-Doh® (Hasbro, Pawtucket, RI, USA), is used to shape the uterus (Figure 7). A longitudinal trench is left on top of the fundus, where the fallopian tubes are placed and secured. A segment of pig bowel is cut longitudinally and trimmed to a desirable size, approximately 2 or 3 cm wide. One or more chickpeas, each representing an ectopic pregnancy, are positioned at its center. More than one pea can be placed in the model to allow for repeated practice. The top portion of the bowel is folded in order to cover the chickpea(s) and the opposite walls of the bowel strip are sutured together using a running stitch (with 2-0 silk on a straight needle). The end of the tube that is thereby created is placed in the trench on the body of the plasticine uterus. The model is then placed on a grounding cautery pad inside a plastic container that is lined with a towel. This model is then placed in the box trainer.

This module is for more advanced learners. Using this simple model, the residents learn laparoscopic management of an ectopic pregnancy. Appropriate task-related GRS (Table 2) and checklists (Table 6) are used for both formative and summative feedback. The checklist is adapted from Larsen, et al. [18].

**Table 6:** Task-Specific Checklist: Laparoscopic Salpingectomy Adapted from Larsen, et al.

Item		Not Done or Incorrect	Done Correctly
1	Selects appropriate instruments (graspers, bipolar diathermy, scissors, rinse/suction, bag)	0	1
2	Starts the video recording	0	1
3	Inserts instruments, grasper in lateral trocar, other instruments in medial trocar	0	1
4	Identifies the anatomy	0	1
5	Operates from centre towards lateral	0	1
6	Uses grasper in right hand and grasps the Fallopian tube	0	1
7	Uses bipolar grasper in left hand and uses diathermy on salpinx and mesosalpinx	0	1
8	Starts close to tubal corner of uterus	0	1
9	Shifts bipolar grasper to scissors in left trocar and cuts the coagulated tissue close to the Fallopian tube	0	1
10	Continues alternated use of bipolar grasper and scissors to remove Fallopian tube. Uses instruments in the trocars providing the most appropriate access to the tissue	0	1
11	Takes care not to use diathermy on the ovary and the supplying artery and other non-target tissue	0	1
12	Uses bag or grasper to remove the dissected tissue and rinse/suction device to clean up blood	0	1
13	Uses bipolar grasper to coagulate any remaining bleeding vessels/tissue	0	1
14	Stops video recording	0	1
Maximum total score, Total score			(14)

### Module 7: Ovarian cystectomy

**Figure 8:**
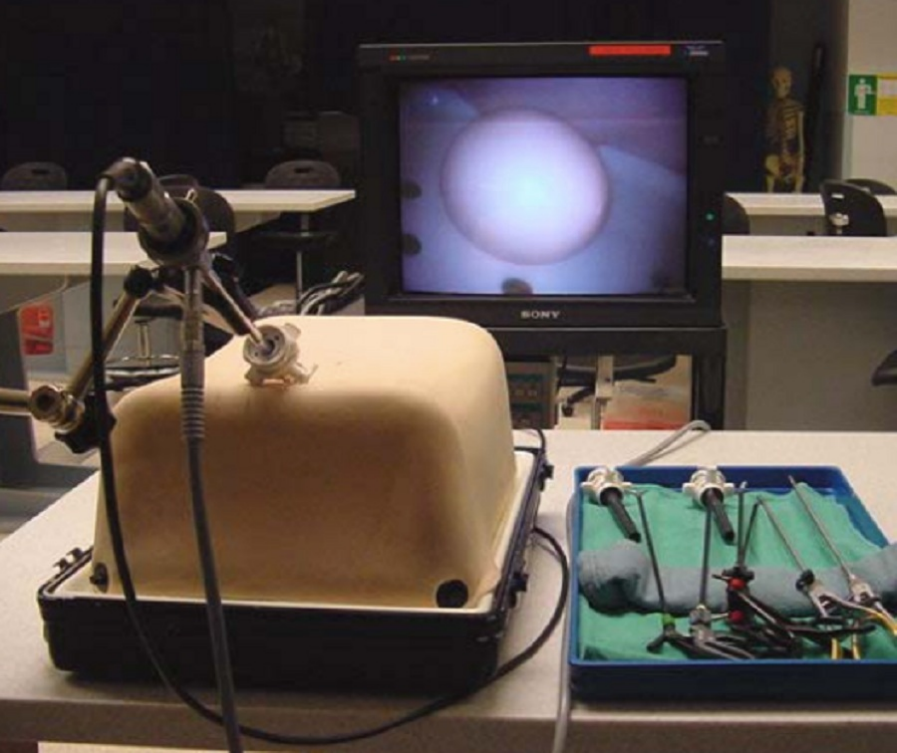
Ovarian cystectomy lap trainer.

The residents' learning objectives here are to develop specific skills related to ovarian cystectomy. To make the ovarian model, a balloon filled with honey was placed inside a larger balloon inflated with air to form a sphere approximately 4 cm in diameter. The two balloons were tied off separately. The model was attached to the box trainer with a crocodile clamp - suction cup assembly. Finally, the model was covered with a synthetic skin (Figure 8).

This module is again for advanced learners. Using this model, the residents learn laparoscopic management of cholecystectomy. Appropriate task-related GRS (Table 2) and checklists (Table 7) are used for feedback.

**Table 7:** Task-Specific Checklist: Laparoscopic Ovarian Cystectomy

Item		Not Done or Incorrect	Done Correctly
1	Inspects the pelvis and upper abdomen	0	1
2	Selects atraumatic graspers	0	1
3	Stabilizes ovary	0	1
4	Selects laparoscopic scissors	0	1
5	Introduces scissors under direct visualization	0	1
6	Incises the ovarian cortex over the ovarian cyst	0	1
7	Does not puncture the cyst	0	1
8	Bluntly dissects the cyst wall free from the overlying ovarian cortex	0	1
9	Using graspers, provides traction and counter-traction until the entire cyst wall is free	0	1
10	Always keeps instruments in view while in the abdomen	0	1
11	Does not overshoot the target more than 20% of the time	0	1
12	Irrigates the area	0	1
13	Inspects for hemostasis	0	1
14	Follows removal of cyst from the abdomen with the camera	0	1
Maximum total score, Total score			(14)

## Discussion

The advantages of laparoscopic surgery over open surgery are the same in Haiti as they are in Canada. Laparoscopic surgery avoids large open wounds and incisions, thereby reducing postoperative discomfort and the need for analgesia. Fine laparoscopic instruments cause little tissue damage and blood loss. Moreover, the rate of postoperative complications is generally lower, especially those related to the wound (e.g. infection, dehiscence, herniation,etc.). The procedure is performed within the body cavity, which reduces most of the handling associated with open procedures. This reduces postoperative adhesions, infections, and keloids. Cumulatively, these benefits help to decrease the recovery period and associated hospital stay [19].

For these reasons, laparoscopic surgery is desirable in developing countries like Haiti. However, both limited access to training and financial restraints impede learning and disseminating the procedure. We describe a locally developed box trainer and accompanying educational curriculum that can help to implement a sustainable laparoscopic skills training program. This may ultimately prepare JUH surgical and obstetrical residents to train and work at other hospitals throughout Haiti where laparoscopy is already being performed. Both the trainer and its curriculum should be piloted and evaluated in order to assess its clinical feasibility. 

## Conclusions

Laparoscopic surgery offers numerous advantages. However, in developing countries, there are logistical and financial barriers to its use. The purpose of this technical report was to provide surgical educators with a cost-efficient and locally developed simulation teaching tool. Comprised of seven locally developed modules and validated assessment tools, it may help mitigate barriers to laparoscopic training and eventually help facilitate its use in developing countries. A future pilot and assessment of both the tool and its accompanying curriculum may help to establish its clinical utility.
